# Optimisation of Tet-On inducible systems for Sleeping Beauty-based chimeric antigen receptor (CAR) applications

**DOI:** 10.1038/s41598-020-70022-0

**Published:** 2020-08-04

**Authors:** S. M. Ali Hosseini Rad, Aarati Poudel, Grace Min Yi Tan, Alexander D. McLellan

**Affiliations:** Department of Microbiology and Immunology, University of Otago, Dunedin, Otago 9010 New Zealand

**Keywords:** Biotechnology, Immunology, Molecular biology

## Abstract

Regulated expression of genetic elements that either encode polypeptides or various types of functional RNA is a fundamental goal for gene therapy. Inducible expression may be preferred over constitutive promoters to allow clinician-based control of gene expression. Existing Tet-On systems represent one of the tightest rheostats for control of gene expression in mammals. However, basal expression in absence of tetracycline compromises the widespread application of Tet-controlled systems in gene therapy. We demonstrate that the order of P2A-linked genes of interest was critical for maximal response and tightness of a chimeric antigen receptor (CAR)-based construct. The introduction of G72V mutation in the activation region of the TetR component of the rtTA further improved the fold response. Although the G72V mutation resulted in a removal of a cryptic splice site within rtTA, additional removal of this splice site led to only a modest improvement in the fold-response. Selective removal of key promoter elements (namely the BRE, TATA box, DPE and the four predicted Inr) confirmed the suitability of the minimal CMV promoter and its downstream sequences for supporting inducible expression. The results demonstrate marked improvement of the rtTA based Tet-On system in Sleeping Beauty for applications such as CAR T cell therapy.

## Introduction

Inducible-gene expression is one of the most sought-after elements of synthetic gene regulation systems. Engineering mammalian cells to express proteins or RNA in an inducible fashion offers opportunities for the development of safe cellular-based therapies to treat a wide spectrum of inborn and acquired diseases. Compared to prokaryote genetic systems, the development of tight, inducible gene expression in eukaryote cells has been challenging^1–3^. Unlike prokaryotes, mammalian genetic control is not usually mediated by single or oligo-component regulators, but rather by multiple transcription factors that bind to both promoters, as well as often distant enhancer regions located on different chromosomes. Moreover, both promoters and enhancers may be regulated by epigenetic control mechanisms, and the site of transgene insertion in the genome influences the response profile of transgenes^4^. Tet-On systems utilise a mutant TetR component that binds to tetracycline response elements (TRE) in the presence of tetracycline, or its stable analogue doxycycline^5^. To activate transcription, fusion of the herpes-simplex VP16 transcriptional activator to the C-terminus of the mutant TetR, recruits generalised transcription factors, as well as RNAP II to initiate gene transcription. Modified tetracycline-inducible systems represent the most widely used inducible system in eukaryotic systems, from yeast to human cells^5^. The potential exists for drug inducible systems to be used in cell-based immunotherapy to control the expression of genes or other sequences of interest (GOI). Although 10^3^ to 10^6^-fold induction of gene expression with tetracycline-based control systems has been reported, basal expression in the absence of inducer can result in undesirable GOI expression^5^. In vivo use would be compromised by such leakiness, particularly if the GOI was involved in T cell survival, or resistance to apoptosis. Unfortunately, compared to Tet-Off systems, Tet-On systems are less sensitive to tetracycline and generally exhibit a higher level of basal expression in the absence of induction^6^. On the other hand, Tet-Off systems are slow to respond to withdrawal of tetracycline and this may be compounded by sequestration of tetracycline in vivo, especially within bones^2,7^.

The Sleeping Beauty (SB) transposon system was developed from extinct Salmonid transposons awoken after 10 million years of inactivity through consensus-based correction of accumulated mutations^8^. Compared to retroviral-based insertions into transcriptional units and their regulatory regions, SB vectors insert almost randomly into TA-sites throughout the genome. This property minimises deleterious integrations and helps maintain constitutive or inducible gene expression^9,10^. SB-based vectors carry a GOI along with optional markers or selection elements flanked by inverted terminal repeats (ITRs)^9–12^. Although the transposase has been re-engineered to enhance activity^12,13^, a lower-activity SB-transposase is preferred for human clinical trials to minimise the incidence of multiple genome integrations. Along with piggy bac transposase systems, SB transposase systems have been used in CAR T cell therapy trials for B cell malignancies^14,15^. To expand the utility of SB-based vectors to express a CAR together with additional GOI under drug-control, we revisited the SB-based Tet-On system, through: (1) alterations in the placement of genes within the P2A-linked CAR cassette, (2) the introduction of a G72V mutation in rtTA-M2 – previously only described for yeast Tet-On control^16^ (3) the placement of rtTA under auto-regulatory control, (4) the removal of cryptic splicing sites, and (5) modifications of the proximal promoter. To test the induction of the Tet-On system, we expressed myeloid leukaemia cell differentiation (Mcl-1) as a GOI involved in T cell survival and resistance to apoptosis.

## Results

### The rtTA location within a multi-gene cassette influences responsiveness of the Tet-On system

**Figure 1:**
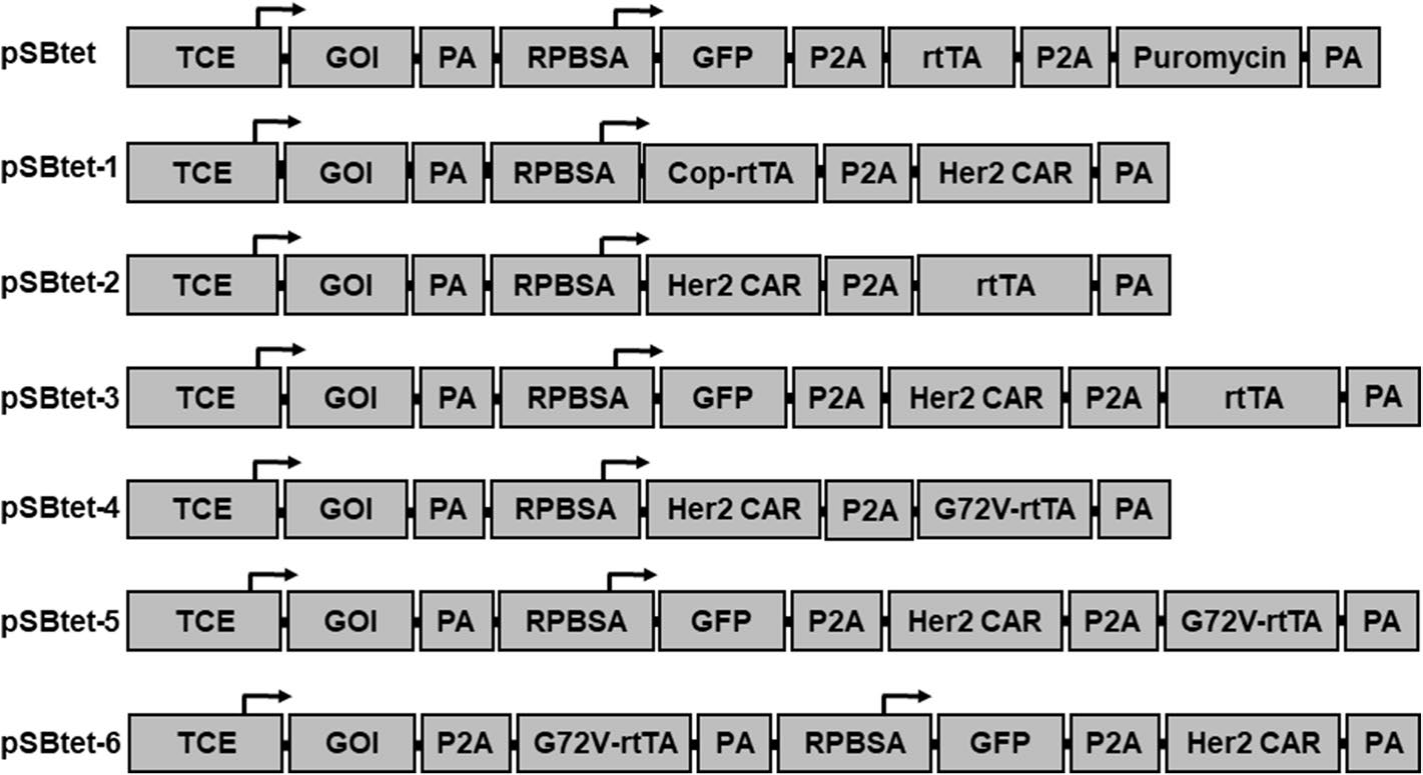
Schematic illustration of SB-based Tet-On systems used in this study. Constructs derived from original pSBtet-GP developed by Kowarz et al. TCE: tet-responsive promoter/ GOI: gene of interest (Mcl-1 or firefly luciferase); PA: polyadenylation site; P2A: 2A self-cleaving; rtTA-M2: reverse tetracycline-controlled transactivator; RPBSA: constitutive promoter comprised of the Rpl13a core promoter and exon 1, plus additional exon and intron elements from Rpl41.

We reasoned that placing a codon-optimised rtTA-M2 gene proximal to the RPBSA promoter (pSBtet-1) should result in robust rtTA-M2 expression and therefore tight control of inducible gene expression (Fig. 1).

**Figure 2:**
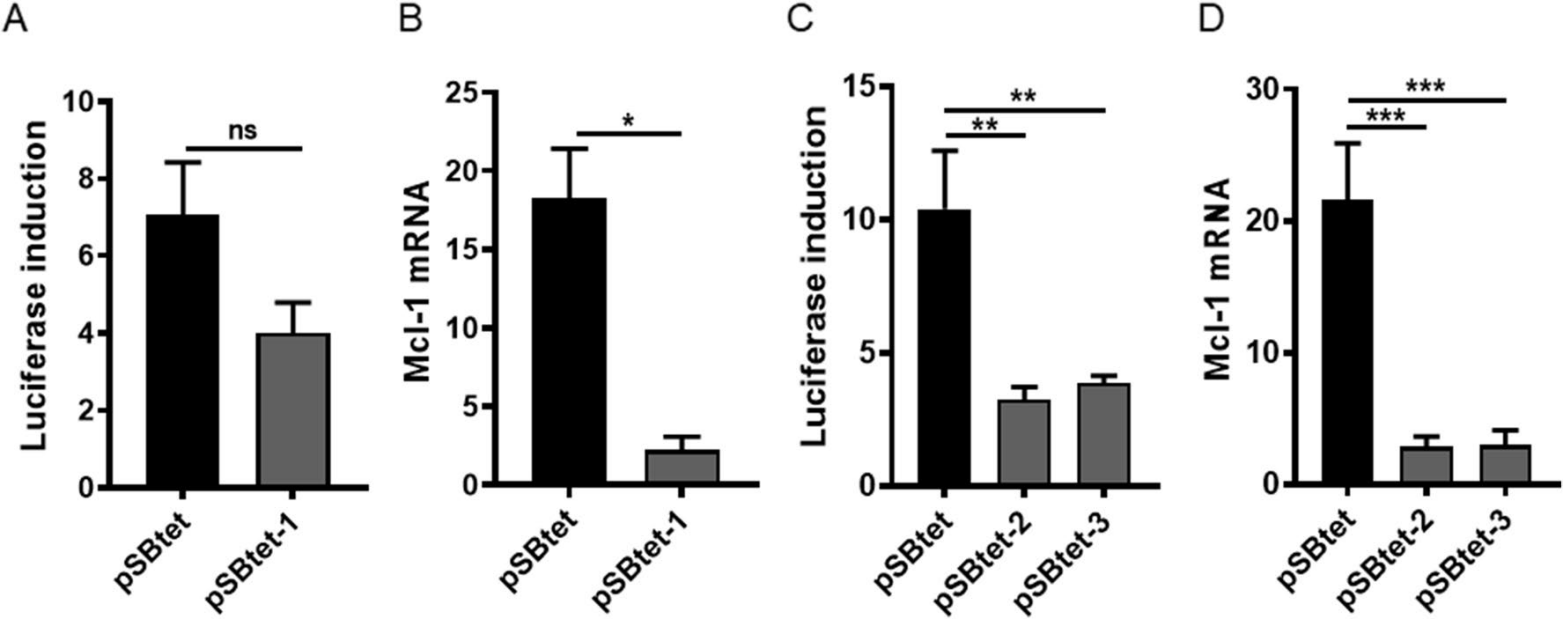
Locating a commercially-codon optimised rtTA-M2 (cop-rtTA; pSBtet-1) proximal to RPBSA increases the leakiness of TCE promoter as measured by (**A**) luciferase fold induction and (**B**) qPCR for Mcl-1 mRNA. Relocating the unmodified rtTA-M2 distal to the RPBSA in (pSBtet-2 &-3) did not improve the basal expression measured by (**C**) luciferase fold induction and (**D**) qPCR for Mcl-1 mRNA. Experiments were carried out 96 h post-transfection. Statistical analysis: (**A**, **B**) two-tailed t-test, (**C**, **D**) one-way ANOVA test with Bonferroni post-test correction (*P < 0.05, **P < 0.01, ***P < 0.001).

Surprisingly, this setting led to a decrease in both the fold-expression of luciferase and Mcl-1 mRNA (Fig. 2A,B). It has previously been reported that expression of rtTA-M2 by strong promoters compromises inducible expression^16,17^. We therefore relocated the original rtTA-M2 sequence distal to the RPBSA and downstream from either one (pSBtet-2) or two (pSBtet-3) additional GOI. However, inducibility of the GOI was still poor (Figs. 1, 2C,D).

### Introduction of a G72V mutation in rtTA-M2 enhances the tightness of the Tet-On system

**Figure 3:**
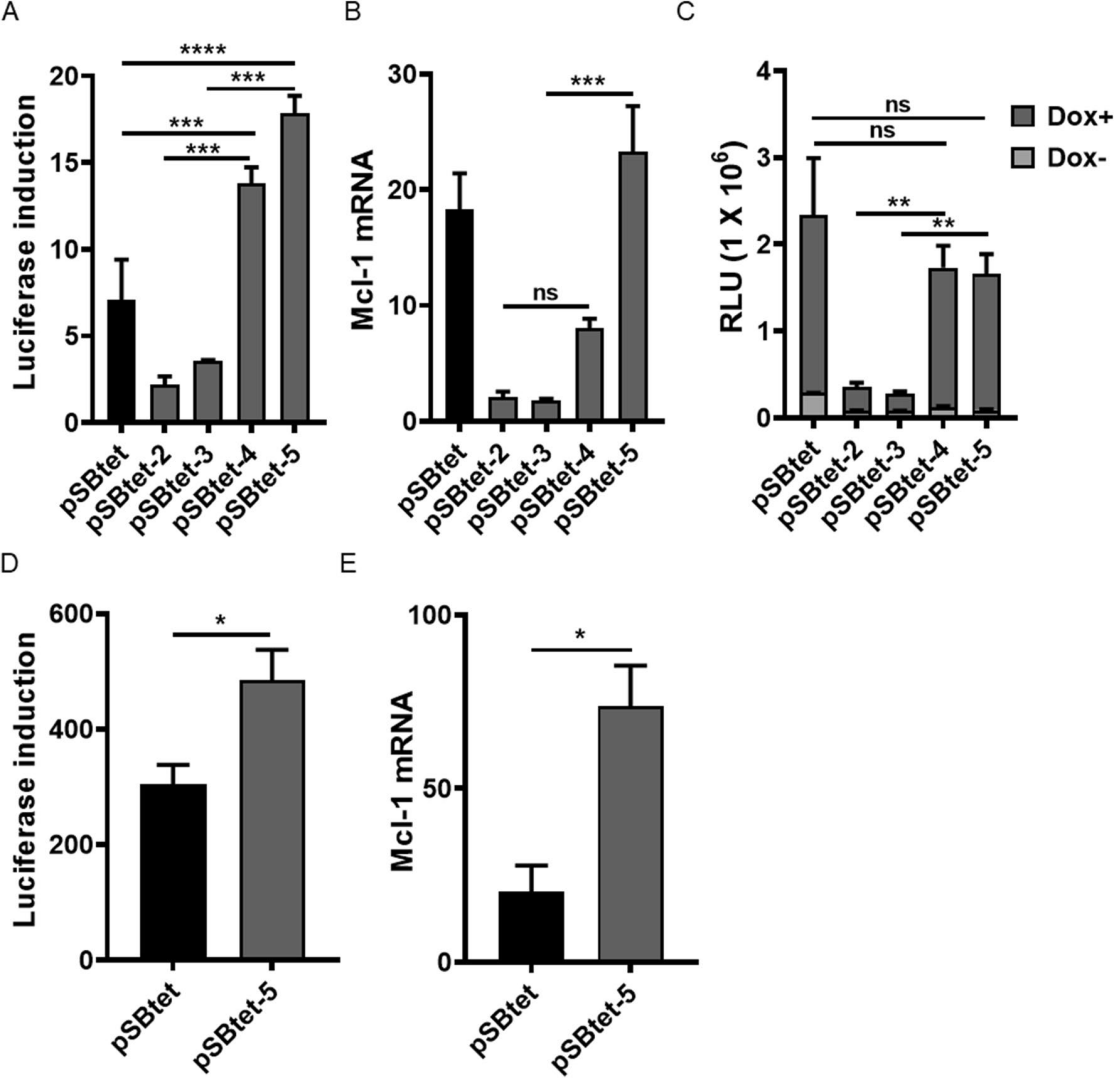
Introducing the G72V mutation into pSBtet-2 & -3 and generation of pSBtet-4 & -5. The efficacy of G72V-rtTA SB based Tet-On system was measured at 96 h by (**A**) luciferase fold induction and (**B**) qPCR for Mcl-1 mRNA. (**C**) The G72V mutation restores the inducibility of pSBtet-2 and pSBtet-3 upon doxycycline induction. (**D**, **E**) Confirmation of the improvement of SB-based Tet-On system after two weeks passaging, as quantified by luciferase fold induction and qPCR for Mcl-1 mRNA. Statistical analysis: (**A**–**C**) one-way ANOVA test with Bonferroni post-test correction, (**D**, **E**) two-tailed t-test (*P < 0.05, **P < 0.01, ***P < 0.001, ****P < 0.0001).

Roney et al.^16^ reported that a GGG to GTG (G72V) missense mutation in rtTA mitigated basal gene expression in the absence of an inducer in *S. cerevisiae* clones. Because of the similarity of transcriptional machinery amongst eukaryote cells, we reasoned that this approach may give similar results in human cells. The G72V mutation was next introduced into pSBtet-2 and pSBtet-3 to create the pSBtet-4 and pSBtet-5 constructs (Fig. 1). The G72V mutation in rtTA-M2 decreased the background expression of TCE promoter in the absence of doxycycline at both the mRNA and protein level (P < 0.001, Fig. 3A,B). The G72V mutation also restored the maximal expression of pSBtet-2 and pSBtet-3 constructs following induction with doxycycline (Fig. 3C). As previously reported, G72V-rtTA-M2 appeared less sensitive to doxycycline compared to original rtTA-M2, though this was not statistically significant (Fig. 3C, P > 0.9)^16^. A similar pattern of results was obtained after two weeks passage of cells to ensure stable integration of the pSBtet-5 (Fig. 3D,E). Note, the GFP expression of the transfected cell lines dropped from ~ 90 to ~ 70% after two weeks of culture, most likely due to a shift from transient gene expression to that from integrated cassettes.

### Investigation of autoregulatory strategy with G72V variant

**Figure 4:**
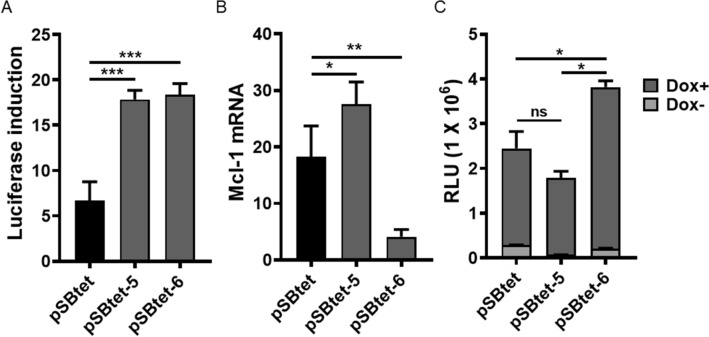
pSBtet-6 autoregulatory system showed superior regulation to pSBtet with (**A**) luciferase fold induction, but (**B**) higher background expression at the mRNA level for Mcl-1. (**C**) Comparison of maximal expression of luciferase in pSBtet-6 versus constitutive expression of G72V (psBtet-5) after induction with doxycycline. pSBtet-6 showed higher sensitivity, but higher background expression in the absence of doxycycline compared to pSBtet-5. Experiments were carried out 96 h post-transfection. Statistical analysis: one-way ANOVA test with Bonferroni post-test correction (*P < 0.05, **P < 0.01, ***P < 0.001).

We next investigated possible improvements in inducibility of multiple GOIs using positive feedback control in an autoregulatory cassette. Autoregulation can improve tetracycline-regulation in a retroviral vector^18^ and in a bi-directional^19^ or uni-directional lentiviral vector^7^. The bi-directional approach appears tight in transient transfection, however, high background was detected when cells were stably transduced^19^. Therefore, we utilised a uni-directional strategy with a P2A sequence in place of an Internal Ribosome Entry Site (IRES) sequence, to allow expression of GOI-P2A-G72VrtTA under TCE promoter (pSBtet-6,Fig. 1). We speculated that the leaky expression of the TCE would still allow sufficient levels of G72V-rtTA inducer to respond to doxycycline stimulation. Although the positive feedback system resulted in tight expression at the protein level (Fig. 4A), as previously reported^7,18,19^, the system was leaky at the mRNA level for Mcl-1 (Fig. 4B). Basal expression of luciferase in pSBtet-6 was lower than for pSBtet (P < 0.05) and showed a higher response upon induction (P < 0.05, Fig. 4C). Compared to the constitutive expression of G72V-rtTA, the autoregulatory system showed greater sensitivity to doxycycline induction (P < 0.05; Fig. 4C), although the basal expression was higher (P = 0.0066).

### Removing cryptic alternative splice sites within rtTA reduces the background expression

**Figure 5:**
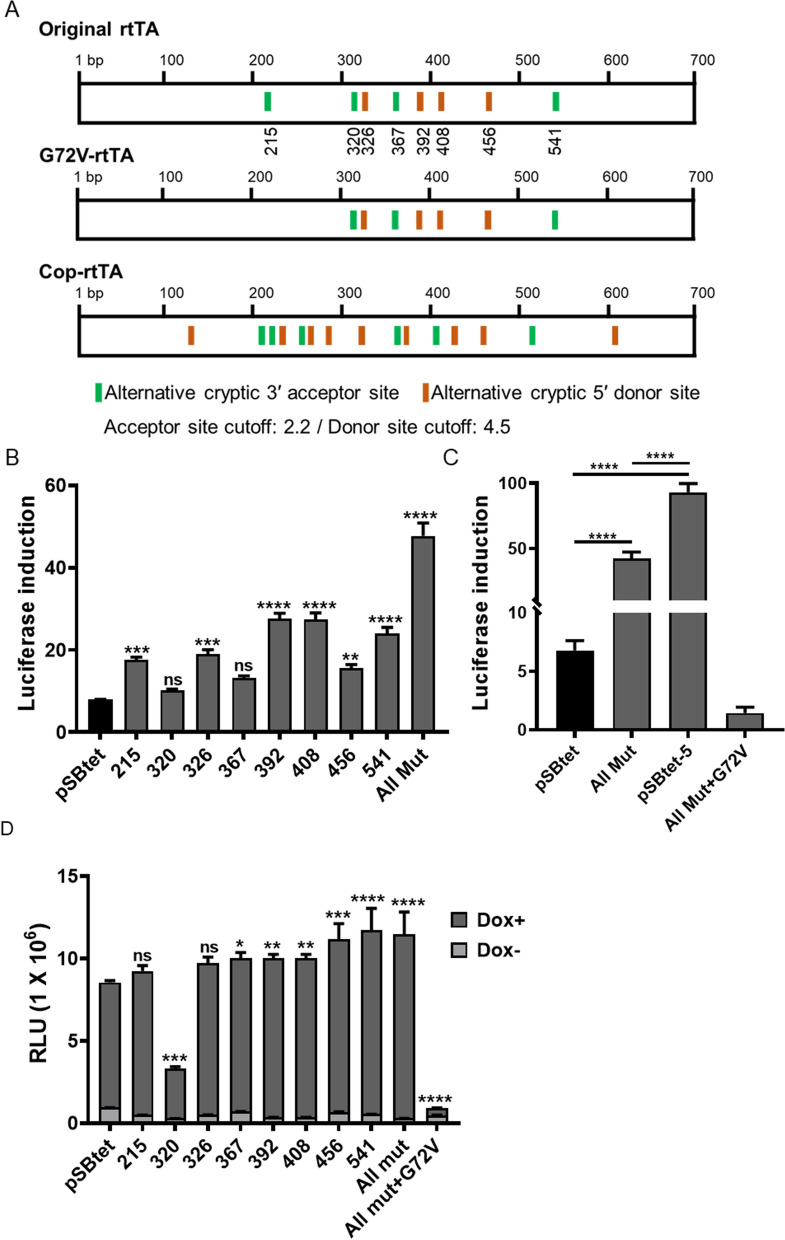
Investigation of potential cryptic splice sites within rtTA. (**A**) Predicted cryptic alternative splice sites within (top): original rtTA-M2, (middle): G72V-rtTA-M2 and (below): commercially codon optimised (cop)-rtTA-M2 using ASSP program. The default cut-off values of the ASSP program was used. The cut-off 2.2 for acceptor sites and 4.5 for donor sites have shown to correctly predict 75 to 80% of cryptic splice sites (Wang M and et al. 2006). (**B**) Removing the eight potential cryptic splice sites alone, or combination of all eight, improved the tightness of Tet-On system. (**C**) Comparison of the fold induction of pSBtet-5 with removing all cryptic splice sites in pSBtet-5. Combining G72V mutation with the eight cryptic splice sites removed, resulted in a non-responsiveness Tet-On system (**D**) Induction of luciferase expression in mutated rtTA-M2 proteins upon doxycycline induction. The missense E107Q mutation at position 320 bp showed lower induction. Experiments were carried out 96 h post-transfection. Statistical analysis: one-way ANOVA test with Bonferroni post-test correction (*P < 0.05, **P < 0.01, ***P < 0.001, ****P < 0.0001).

Since the first description of the eukaryotic Tet-On inducible system, most optimisation studies have focused on rtTA mutations: for example, the removal of cryptic splice sites (flanking amino acids 8–144) in the TetR sequence^20,21^. Using recently-developed software^22^, we identified eight additional potential cryptic splice sites within the coding region of rtTA-M2 (Fig. 5A and Table S1). Because the G72V mutation resulted in the loss of a cryptic splice site at position 215 (Fig. 5A and Table S2, we determined if the success of the G72V mutation was due to the removal of the potential cryptic splice site at 215. These cryptic splice sites are located in two regions of rtTA; one in a surface residue (215 nt and seven in the dimerisation domains (320 nt, 326 nt, 367 nt, 392 nt, 408 nt, 456 nt and 541 nt; Fig. 5A, Figure S1). We therefore removed all eight cryptic splices sites by silent or conserved missense-mutations in the pSBtet construct (Table 1).

**Table 1:** Putative cryptic acceptor and donor splice sites within rtTA. Dinucleotide splice sites (AG or GT) are highlighted in bold and mutated sequences are underlined.

Name	Type	Original sequence	Mutated sequence	Mutation type
215	Acceptor	CCC CTG GA**A** **G**GC GAG TCA Pro Leu Glu Gly Glu Ser	CCC CTG GA**T** **G**GC GAG TCA Pro Leu Asp Gly Glu Ser	Missense (conservative)
320	Acceptor	CCA AC**A** **G**AG AAA CAG TAC Pro Thr Glu Lys Gln Tyr	CCA AC**A** **C**AA AAA CAG TAC Pro Thr Gln Lys Gln Tyr	Missense (conservative)
326	Donor	CCA ACA GAG AAA CA**G** **T**AC Pro Thr Glu Lys Gln Tyr	CCA ACA GAG AAA CA**A** **T**AC Pro Thr Glu Lys Gln Tyr	Silent
367	Acceptor	TGT C**AG** CAA GGC TTC TCC Cys Gln Gln Gly Phe Ser	TGT C**AA** CAA GGC TTC TCC Cys Gln Gln Gly Phe Ser	Silent
392	Donor	AAC GCA CT**G** **T**AC GCT CTG Asn Ala Leu Tyr Ala Leu	AAC GCA TT**A** **T**AC GCT CTG Asn Ala Leu Tyr Ala Leu	Silent
408	Donor	TCC GCC **GT**G GGC CAC TTT Ser Ala Val Gly His Phe	TCC GCC **AT**C GGC CAC TTT Ser Ala Ile Gly His Phe	Missense (conservative)
456	Donor	GAG CAT CAA **GT**A GCA AAA Glu His Gln Val Ala Lys	GAG CAT CAA **GT**G GCA AAA Glu His Gln Val Ala Lys	Silent
541	Acceptor	GAC CGG C**AG** GGA GCC GAA Asp Arg Gln Gly Ala Glu	GAC CGG C**AA** GGA GCC GAA Asp Arg Gln Gly Ala Glu	Silent

The removal of six cryptic splice sites modestly enhanced the tightness of Tet-On system 7.7–19.6 fold compared with original rtTA (P < 0.001, Fig. 5B). The remaining two mutations at position 320 (~ twofold, P = 0.8) and 367 (~ fivefold, P = 0.08) did not significantly affect Tet-On performance. The mutation at position 320 produced E106Q, while 367 (Q122) was a silent mutation (Table 1). It is possible these two splice sites are weak 5′ acceptor splice sites which are only used if other competing splice sites are removed^23^. Indeed, positions 320 and 367 have low score and confidence which represent strength and the probable occurrence of a splice site, respectively (Table S1). Combining all mutations together, improved the leaky background of the Tet-On system ~ 40 fold compared to original rtTA (P < 0.0001, Fig. 5B). However, superior results were still seen with G72V mutation (Fig. 5C).

**Figure 6:**
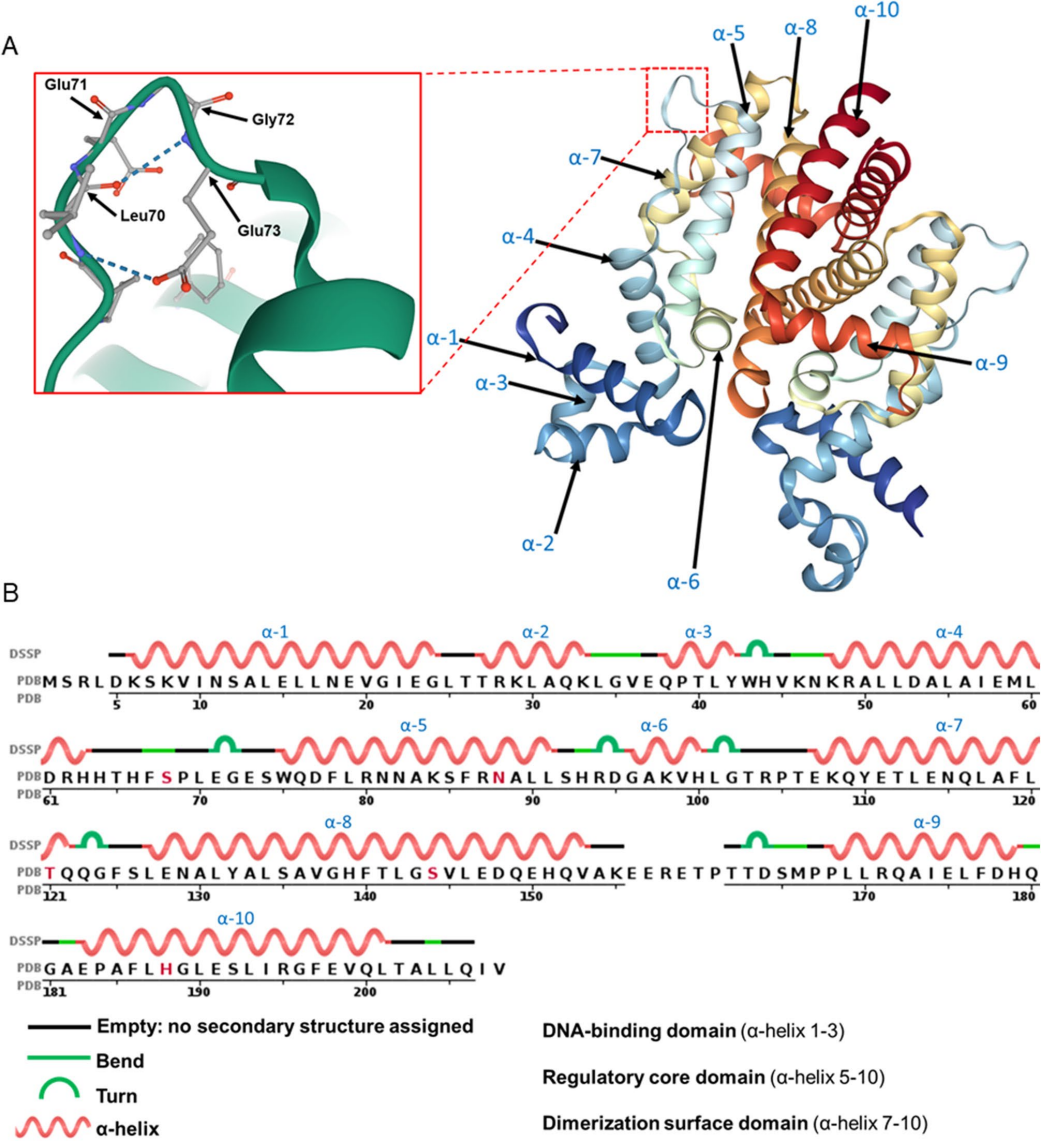
(**A**) Secondary structure of TetR obtained from protein data bank (PBD) with focus on the E71 and G72 (highlighted by red box) that form a turn between α-helix-4 and 5. Mutating both amino acids may cause a conformational change in rtTA. (**B**) Annotation of TetR protein sequence and position of the ten α-helices.

Surprisingly, combining the G72V mutation and removing all cryptic splice sites abolished responsiveness and inducibility of Tet-On system (Fig. 5C,D). There are four altered amino acid positions within rtTA-M2 that result in a reverse activator phenotype, as compared to the original TetR: E71K, D95N, L101S and G102D^2^. In TetR, E71 is a surface residue amino acid, D95 connects the DNA reading head to the core domain, while L101 and G102 are crucial for dimerisation and the tetracycline response, respectively^2^. In TetR the E71 and G72 amino acids create the turn between α-helix-4 and 5 (Fig. 6). This region bridges the DNA binding domain to the tetracycline binding domain and the combination of both the E71K and G72V mutations might destroy the structure of this critical turn, causing a loss of rtTA-M2 activity. This may also explain the drop in tetracycline-induction observed with the position 320 mutant (E107 to Q107, Fig. 5D), since this residue is close to a 'high sensitivity region' (Figure S2)^21,24–26^. It is possible that the G72V mutation affected the secondary structure of rtTA^16^, rather than simply removing a cryptic splice site. It is interesting to note that further commercial algorithm-mediated codon optimisation of rt-TA-M2 attempted in pSBtet-1 (see Fig. 2) re-introduced 13 cryptic alternative splice sites with high score and confidence (Fig. 5A, Table S3) within the rtTA coding region. This may have contributed to the poor performance of the first pSBtet-1 construct analysed, since cryptic splice sites might be associated with poor performance of Tet-On systems^20,21^ (Fig. 2A,B).

### Dissection of the TCE proximal promoter

**Figure 7:**
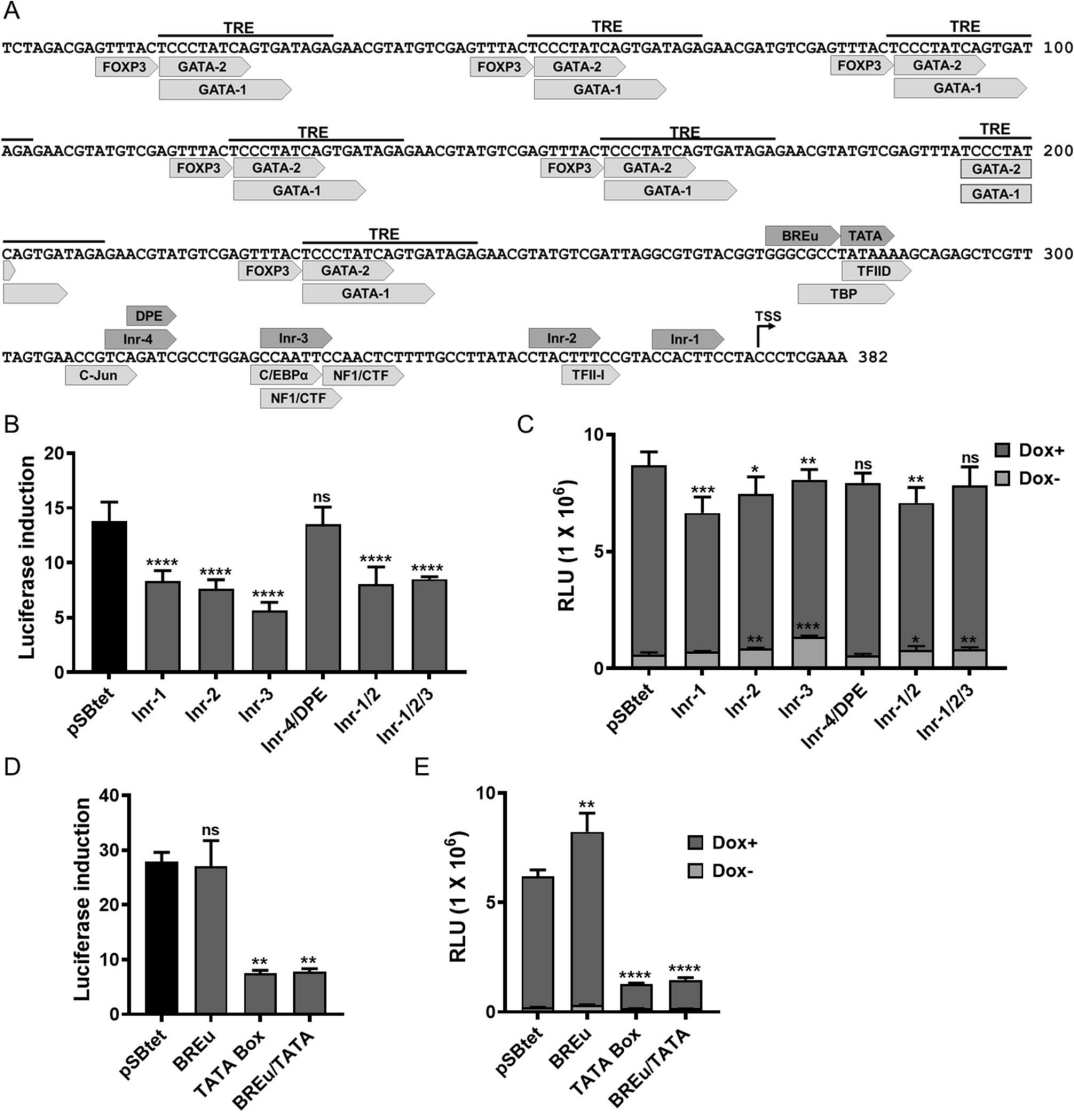
Investigation of core promoter elements in the proximal region of TCE promoter. (**A**) Annotated TCE promoter sequence for core promoter elements and TF binding sites. (**B**, **C**) Deleting elements downstream of TATA box including Inr-1, -2, -3, -4 and DPE in single or combination form increases leaky expression of TCE as well as reduction in TCE promoter induction (**D**, **E**) The effect of removing TATA box and BREu on luciferase induction with or without induction by doxycycline. Removing BREu does not affect the tightness of TCE promoter, whereas TCE showed to be sensitive over loss of TATA box. Only removing BREu improved the inducibility of TCE promoter, without increasing the basal expression. Experiments were carried out 96 h post-transfection. Statistical analysis: one-way ANOVA test with Bonferroni post-test correction (*P < 0.05, **P < 0.01, ***P < 0.001, ****P < 0.0001). Inr: initiation repeats; BRE: B recognition element (BRE); DPE: downstream promoter element; TSS: transcriptional start site.

Modification of the minimal CMV promoter can affect TCE promoter performance^27^. Removing elements downstream of the TATA box can reduce the maximal expression, whereas deleting the upstream elements can decrease the leakiness^27^. We therefore revisited the design of pTIGHT to ensure optimal performance in our setting. Core promoter elements were identified using the YAAP program (Fig. 7A). It is possible that the presence of alternative initiator element (Inr) might lead to a loss of control of the TCE-promoter. We therefore removed these elements in single or combinatorial mutation fashion from pSBtet and monitored the tightness and maximal expression of TCE promoter.

Removal of each, or all, downstream elements of the TATA box (Inr-1, -2, -3 and -4 sites, and the DPE element) markedly decreased the tightness of the TCE promoter (Fig. 7B), and also reduced the maximal expression, as previously reported^27^ (Fig. 7C). Specifically, removing Inr-3 increased the background expression remarkably (P < 0.01). Inr-3 contains two CTF/NF1 binding sites that bind to DNA as dimers^28^ (Fig. 7A). CTF/NF1 is an enhancer-blocker element which specifically blocks the interaction of other enhancers with the promoter^29^. A general explanation would be that deletion of the CTF/NF1 binding site could increase the interaction of neighbouring enhancers to the TCE promoter, resulting in high background.

The only core promoter element found upstream of the TATA box is B recognition element (BREu). As shown in Fig. 7D,E, removing BREu did not improve the tightness and the basal expression of the TCE promoter (P > 0.19) and optimal transcription through the TCE promoter was dependent on the TATA box. However, deleting the BREu site increased the response of the TCE promoter to doxycycline (Fig. 7E, P < 0.01).

## Discussion

Several strategies have been proposed to reduce the leakiness and enhance the inducibility of Tet-On systems, with only some tested in human cells. Such approaches include: (1) increased expression of rtTA using a strong promoter and codon optimisation^21,30,31^, (2) mutation of rtTA to increase binding to doxycycline or DNA^16,20,21,26^, (3) autoregulatory systems^7,18,19,32^, (4) removing a cryptic splice sites in the rtTA coding region^20^, and (5) alteration of the core promoter elements within the proximal region of the TCE promoter ^27^. We revisited these strategies for use in the SB-based Tet-On system in a human cell line for future investigation in CAR T cell therapy.

The introduction of a single mutation G72V, gave the optimal induction results at both mRNA and protein level, as reported in *S. cerevisiae*^16^. Future studies may explore the use of a G72P instead of G72V in our system as a candidate amino acid at position G72, though G72P appeared to result in a small loss in sensitivity, as compared to G72V^16^. It is interesting that independent efforts into the rtTA structure have resulted in distinct amino acid changes in different studies, but with similar outcomes. For example, mutations introduced into the rtTA-M2 gene used here are present in distinct positions, as compared to the original four mutations in rtTA^6,20^. Moreover, introducing sensitivity enhancing (SE) mutations^24–26^ (V9I, F67S, G72P, F86Y, and R171K) could further increase the sensitivity to doxycycline, without increasing the background, as demonstrated in yeast^16^.

Autoregulatory systems have recently generated interest, with both the rtTA and GOI transcribed by a single TCE promoter, using either a bi-directional promoter^19^ or an IRES sequence^7,18,32^. However, our constitutive expression of G72V-rtTA gave tighter expression, but was less sensitive to doxycycline compared to the autoregulatory system. The autoregulatory system may be preferred for controllable expression of a toxic rtTA or toxic GOI in mammalian protein production^32–35^.

Next, our analysis found evidence of cryptic splice sites within an rtTA, a sequence that was previously optimised for mammalian expression by Urlinger et al.^30^ Removing these splice sites reduced the basal expression and further increased the maximal expression. Unfortunately, using the combination of the G72V mutation with all splice sites removed (using predominantly silent, but with two necessarily non-silent, mutations) created a non-responsive system. It appears likely that the combination of the E71Q and G72V mutations disrupted the turn between two critical α-helixes 4 and 5.

It is also noteworthy that different programs identified other possible splice sites that needed to be investigated (Table S4). At least three independent approaches for codon optimisation of rtTA have been reported to enhance Tet-On function^21,30,31^. For example, Urlinger et al. modified the *S. cerevisiae*-developed rtTA-M2 sequence to remove potential hairpin, splice, and endonuclease sites, as well as codon optimising the sequence for use within mammalian systems^21^.

In the proximal region of the TCE promoter, we confirmed that the TATA box was essential for the function of the TCE promoter^27^. Removal of the BREu element increased maximal expression, but did not markedly affect the tightness of the TCE. The deletion of the BRE site might enhance the elongation and / or reduce the TFIIB-rtTA sequestration. BRE plays a role in the preinitiation complex (PIC), leading to the dissociation of TFIIB from the promoter which is necessary for RNA polymerase II to initiate the elongation step^36–38^. Hence, interrupting the BRE-TFIIB interaction may enhance transcription via the enhancement of elongation^39^. Alternatively, direct sequestration VP16 on TFIIB has been reported^40,41^ that may act to reduce VP16-mediated transcriptional activation.

Collectively, our results demonstrate marked improvements to the rtTA-M2 based Tet-On system in a Sleeping Beauty system through the yeast-optimised G72V mutation. The results especially highlight the necessity to investigate the placement of individual GOI and rtTA within an expression cassette. The use of the clinically relevant CAR cassette within this setting offer the possibility to enhance adoptive cell therapy though drug-inducible expression of cell-survival and memory enhancing genes, or death switches to conditionally ablate CAR T cells following the onset of cytokine release syndrome.

## Material and methods

### Plasmid construction and cloning

The Tet-On SB (pSBtet-GP) contains the tetracycline-inducible pTIGHT promoter upstream of two asymmetric SfiI sites for cloning genes of interest (GOI), with a downstream RPBSA promoter driving GFP-P2A-rtTA-P2A-puromycin. pSBtet-GP and the SB-transposase vector (pCMV(CAT)T7-SB100) were purchased from Addgene. The pTIGHT promoter is a derivative of (Ptet-14) with shorter spacer (16–17 bp) sequences^27^ between the TRE and the minimal CMV promoter (see Fig. 7)^9,27,42^. To generate the modified SB plasmids, a multiple cloning site (MCS) with Bsu36I and BstBI sites was cloned into pSBtet to remove GFP-P2A-rtTA-P2A-puromycin to create pSBtet-MCS. The codon optimised rtTA-M2 gene and FRP5 scFv Her2-CAR^43^ were synthesised as gene blocks (IDT Singapore) and cloned into pSBtet MCS to create pSBtet-1. Other plasmids were generated by splicing by overlap extension (SOE) PCR to fuse the original rtTA2S-M2 (rtTA)^9,21^, GFP and Her2CAR in different combinations as illustrated in Fig. 1. Mutations into rtTA was introduced using inverse or SOEing PCR. Codon optimised-mouse Mcl-1 (Cop-Mcl-1) was synthetised as a gene block (IDT) with SfiI overhangs to replace the Firefly luciferase gene in the pSB-tet constructs. Both the Mcl-1 and firefly luciferase genes were used as GOI in this study. To modify the core promoters elements (Fig. 5A), the proximal promoter of TCE was PCR amplified from pSBtet and then subcloned into a pUC19 vector (Addgene) using conventional restriction fragment ligation method with EcoRI and NcoI enzymes. Inverse PCR with primers carrying point mutations were used to change the core promoter elements. Finally, each of the modified fragments were PCR amplified from pUC19 and cloned back to pSBtet using PshAI and NcoI restriction sites. To alter the cryptic splice sites within rtTA (Table 1), rtTA was sub-cloned into PUC19 and mutations introduced using inverse PCR.

### Bioinformatics analysis

Analysis of the TCE proximal promoter for core promoter elements, including the initiation repeats (Inr1, 2, 3 and 4), TATA box, B recognition element (BRE) site and downstream promoter element (DPE), was carried out using YAPP Eukaryotic Core promoter predictor. TF binding sites were predicted using AliBaba 2.1^44^ and PROMO^45,46^ programs. The transcriptional start site (TSS) was predicted as reported previously for the minimal CMV promoter^47^. Screening of rtTA for cryptic splice sites was carried out using Alternative Splice Site Predictor (ASSP) software and Human Splicing Finder (HSF)^22,48^. The protein structure of TetR and the prediction of secondary structure were obtained from Protein Data Bank (PBD).

### Cell culture and transfection

The human embryonic kidney 293 (HEK293; ATCC CRL-1573) cell line was cultured in high glucose Dulbecco’s Modified Essential medium (DMEM) supplemented with tetracycline-free 10% foetal bovine serum (FBS; Pan Biotech), Pen-Strep (100 U/mL penicillin and 100 µg/mL streptomycin) (Gibco) at 37 °C with 5% CO_2_. One day prior to transfection, HEK293 cells were cultured in a 24 well plate at 2 × 10^5^ cells/ mL. A ratio of 5:1 (transfer plasmid: transposase) was used to stably transfect HEK293 cell line using Lipofectamine 3,000 (Thermo Fisher) and the medium was replaced at 24 h post transfection. For induction of the TCE promoter, at 72 h post transfection, cells from each well were detached and divided into four wells in a 96-well plate. Two wells were cultured with DMEM containing 5 µg / mL of doxycycline (Sigma), while control wells were maintained with only DMEM, for additional 24 h. Doxycycline-induced and control values for each construct are derived from each independent transfection to eliminate the possibility of different transfection efficiencies between Dox + and Dox- wells. Then, cells were proceed either with qPCR or luciferase assays. For Fig. 3D,E cells were maintained for to two weeks to confirm the consistency of gene-regulation over longer time periods.

### RNA extraction, cDNA synthesis and quantitative PCR (qPCR)

Total RNA was extracted using NucleoSpin RNA Plus kit (Macherey–Nagel, Germany) and cDNA prepared using PrimeScript RT Reagent Kit (Takara Bio, USA). QPCR was carried out using Luna Universal qPCR Master Mix (NEB) in a ViiA 6 Real-Time PCR (Applied Biosystems, Foster City, CA). The comparative CT (2^−ΔΔct^) method was used to analyse the relative expression level of cop-Mcl1, by normalising to β-actin. Primers used for the qPCR reactions were: Mcl1-Fwd: GCA GAA TTG TGA CAC TGA TAA G, Mcl1-Rev: TTT TGT TCT AAC CAA TAC ATC G, β-actin-Fwd: CTT CCT TCC TGG GCA TG, β-actin-Rev: GTC TTT GCG GAT GTC CAC.

### Reporter assay

Luciferase assays were carried out using Pierce Firefly Luc one-step glow assay kit (ThermoFisher #16197) with cells at 10^5^ cells per 100 µL in a 96 well plate. Firefly Luc One-Step Glow assay working solution (100 µL) was added to each well. Cells were incubated at room temperature for one hour before reading with a Varioskan LUX multimode microplate reader (Thermo Fisher, USA). Luciferase data was presented either as relative luminescence units (RLU) or fold change. Fold change was calculated with the following formula:

$${\text{Fold}}\,{\text{change}} = \frac{{{\text{Luciferase}}\,{\text{read}}\,{\text{from}}\,{\text{cells}}\,{\text{treated}}\,{\text{with}}\,{\text{doxycyline}}}}{{{\text{Luciferase}}\,{\text{read}}\,{\text{from}}\,{\text{same}}\,{\text{cells}}\,{\text{untreated}}\,{\text{with}}\,{\text{doxycycline}}}}$$

### Statistical analysis

All data are presented as mean ± standard deviation (SD) and pooled from three independent experiments. Statistical analysis was performed by two-tailed t-test or one-way ANOVA test with Bonferroni post-test correction in GraphPad prism (version 8). The P values of ≤ 0.05 were considered statistically significant (* P < 0.05, ** P < 0.01, *** P < 0.001, **** P < 0.0001). (* P < 0.05, ** P < 0.01, *** P < 0.001, **** P < 0.0001).

## Supplementary information

Supplementary Information.
