# Supplementary material for: Optimisation of Tet-On inducible systems for Sleeping Beauty-based chimeric antigen receptor (CAR) applications
Source: Sci Rep. 2020 Aug 4;10:13125. doi: 10.1038/s41598-020-70022-0 (PMC7403325; doi:10.1038/s41598-020-70022-0)
Supplement: Supplementary file 1 — Supplementary Information. [file 41598_2020_70022_MOESM1_ESM.docx]

**Optimisation of Tet-On inducible systems for Sleeping Beauty-based chimeric antigen receptor (CAR) applications**

Ali Hosseini Rad SM^¶^, Aarati Poudel^¶^, Grace Min Yi Tan^¶^ and Alexander D. McLellan*

^¶^ These authors contributed equally to this work.

Department of Microbiology and Immunology, University of Otago, Dunedin 9010, Otago, New Zealand.

*To whom correspondence should be addressed: Alexander D. McLellan Department of Microbiology and Immunology, University of Otago, Dunedin 9010, Otago, New Zealand; alex.mclellan@otago.ac.nz.

**Table S1**. Potential cryptic alternative splice sites within rtTA coding region by ASSP program.

| **Position** | **Splice site type** | **Sequence** | **Score*** | **Confidence**** |
| --- | --- | --- | --- | --- |
| 215 | Acceptor | cccctggaagGCGAGTCATG | 3.637 | 0.768 |
| 320 | Acceptor | cgcccaacagAGAAACAGTA | 2.567 | 0.552 |
| 326 | Donor | CAGAGAAACAgtacgaaacc | 6.389 | 0.894 |
| 367 | Acceptor | cctgtgtcagCAAGGCTTCT | 3.704 | 0.849 |
| 392 | Donor | AGAACGCACTgtacgctctg | 4.895 | 0.919 |
| 408 | Donor | TCTGTCCGCCgtgggccact | 5.697 | 0.609 |
| 456 | Donor | GGAGCATCAAgtagcaaaag | 5.673 | 0.903 |
| 541 | Acceptor | cgaccggcagGGAGCCGAAC | 5.154 | 0.858 |

**Table S2.** Potential cryptic alternative splice sites within G72V-rtTA coding region by ASSP program.

| **Position** | **Splice site type** | **Sequence** | **Score*** | **Confidence*** |
| --- | --- | --- | --- | --- |
| 320 | Acceptor | cgcccaacagAGAAACAGTA | 2.567 | 0.552 |
| 326 | Donor | CAGAGAAACAgtacgaaacc | 6.389 | 0.894 |
| 367 | Acceptor | cctgtgtcagCAAGGCTTCT | 3.704 | 0.849 |
| 392 | Donor | AGAACGCACTgtacgctctg | 4.895 | 0.919 |
| 408 | Donor | TCTGTCCGCCgtgggccact | 5.697 | 0.609 |
| 456 | Donor | GGAGCATCAAgtagcaaaag | 5.673 | 0.903 |
| 541 | Acceptor | cgaccggcagGGAGCCGAAC | 5.154 | 0.858 |

**Table S3.** Potential cryptic alternative splice sites within Cop-rtTA coding region by ASSP program.

| **Position** | **Splice site type** | **Sequence** | **Score*** | **Confidence*** |
| --- | --- | --- | --- | --- |
| 132 | Donor | GTACTGGCATgtaaaaaaca | 5.127 | 0.917 |
| 214 | Acceptor | tcccttggagGGAGAAAGTT | 3.729 | 0.612 |
| 240 | Acceptor | atttcctcagGAATAACGCC | 3.586 | 0.352 |
| 260 | Donor | AGAGTTTTAGgtgtgcgctc | 8.709 | 0.966 |
| 282 | Acceptor | tgtctcacagAGATGGTGCG | 8.920 | 0.112 |
| 286 | Donor | CACAGAGATGgtgcgaaggt | 6.777 | 0.911 |
| 294 | Donor | TGGTGCGAAGgttcacttgg | 4.538 | 0.936 |
| 326 | Donor | CCGAGAAACAgtatgaaacc | 6.194 | 0.951 |
| 367 | Acceptor | cctgtgccagCAAGGTTTCT | 3.470 | 0.807 |
| 370 | Donor | TGCCAGCAAGgtttctcact | 5.293 | 0.863 |
| 407 | Acceptor | gcactctcagCCGTTGGTCA | 3.323 | 0.932 |
| 428 | Donor | TTACTCTCGGgtgcgtcctc | 7.722 | 0.959 |
| 456 | Donor | GGAACATCAGgtggctaaag | 7.849 | 0.871 |
| 517 | Acceptor | gcttcgccagGCCATTGAAC | 6.456 | 0.275 |
| 605 | Donor | AGCAATTGAAgtgtgagagt | 5.391 | 0.896 |
| 607 | Donor | CAATTGAAGTgtgagagtgg | 8.156 | 0.832 |

***** Score reflects splice site strength and ranges between one to ten.

****** Confidence reflects the probable occurrence of splicing and ranges between zero to one.

**Table S4.** Potential splice sites within rtTA coding region predicted by Human Splice Finder (HSF) program.

| **Position** | **Splice site type** | **Sequence** | **Consensus value (0-100)** |
| --- | --- | --- | --- |
| 7 | Acceptor | agactggacaagAG | 65.22 |
| 9 | Acceptor | actggacaagagCA | 67.25 |
| 22 | Donor | AAAgtcata | 68.79 |
| 47 | Acceptor | tactcaatggagTC | 73.33 |
| 55 | Donor | GGAgtcggt | 69.44 |
| 59 | Acceptor | tcggtatcgaagGC | 76.38 |
| 59 | Donor | TCGgtatcg | 70.52 |
| 72 | Acceptor | cctgacgacaagGA | 73.99 |
| 88 | Acceptor | ctcgctcaaaagCT | 76.86 |
| 100 | Acceptor | ctgggagttgagCA | 68.08 |
| 103 | Acceptor | ggagttgagcagCC | 73.18 |
| 104 | Donor | GAGttgagc | 68.07 |
| 127 | Acceptor | tggcacgtgaagAA | 72.07 |
| 130 | Donor | CACgtgaag | 73.73 |
| 163 | Acceptor | ctgccaatcgagAT | 75.06 |
| 174 | Acceptor | gatgctggacagGC | 80.9 |
| 199 | Acceptor | ttctgccccctgGA | 66.14 |
| 203 | Acceptor | gccccctggaagGC | 77.96 |
| 212 | Donor | AAGgcgagt | 71.05 |
| 216 | Donor | CGAgtcatg | 65.29 |
| 218 | Acceptor | agtcatggcaagAC | 68.31 |
| 222 | Donor | ATGgcaaga | 65.13 |
| 241 | Acceptor | aacaacgccaagTC | 67.3 |
| 249 | Donor | CAAgtcatt | 72.08 |
| 284 | Acceptor | acggggctaaagTG | 68.62 |
| 292 | Donor | AAAgtgcat | 66.34 |
| 308 | Acceptor | cccgcccaacagAG | 85.25 |
| 310 | Acceptor | cgcccaacagagAA | 71.56 |
| 316 | Acceptor | acagagaaacagTA | 70.69 |
| 324 | Donor | ACAgtacga | 72.58 |
| 337 | Acceptor | ctggaaaatcagCT | 75.6 |
| 355 | Acceptor | ttcctgtgtcagCA | 85.01 |
| 359 | Acceptor | tgtgtcagcaagGC | 75.35 |
| 359 | Donor | TGTgtcagc | 75.1 |
| 373 | Acceptor | ttctccctggagAA | 84.47 |
| 390 | Donor | ACTgtacgc | 72.45 |
| 399 | Donor | TCTgtccgc | 66.4 |
| 406 | Donor | GCCgtgggc | 70.8 |
| 430 | Acceptor | tgcgtattggagGA | 77.01 |
| 436 | Acceptor | ttggaggaacagGA | 80.23 |
| 446 | Acceptor | aggagcatcaagTA | 66.28 |
| 449 | Acceptor | agcatcaagtagCA | 68.24 |
| 454 | Donor | CAAgtagca | 66.73 |
| 455 | Acceptor | aagtagcaaaagAG | 67.2 |
| 457 | Acceptor | agactggacaagAG | 65.22 |
| 466 | Donor | GAGgaaaga | 69.07 |
| 501 | Acceptor | cccacttctgagAC | 76.08 |
| 506 | Acceptor | ttctgagacaagCA | 72.82 |
| 514 | Acceptor | caagcaattgagCT | 67.43 |
| 525 | Donor | GCTgttcga | 66.46 |
| 529 | Acceptor | ttcgaccggcagGG | 88.74 |
| 533 | Acceptor | accggcagggagCC | 65.49 |
| 583 | Acceptor | tgtggcctggagAA | 75.88 |
| 589 | Acceptor | ctggagaaacagCT | 75.66 |
| 595 | Acceptor | aaacagctaaagTG | 68.29 |
| 603 | Donor | AAAgtgcga | 76.17 |
| 641 | Acceptor | attttgacttagAC | 76.79 |
| 653 | Acceptor | acatgctcccagCC | 82.52 |


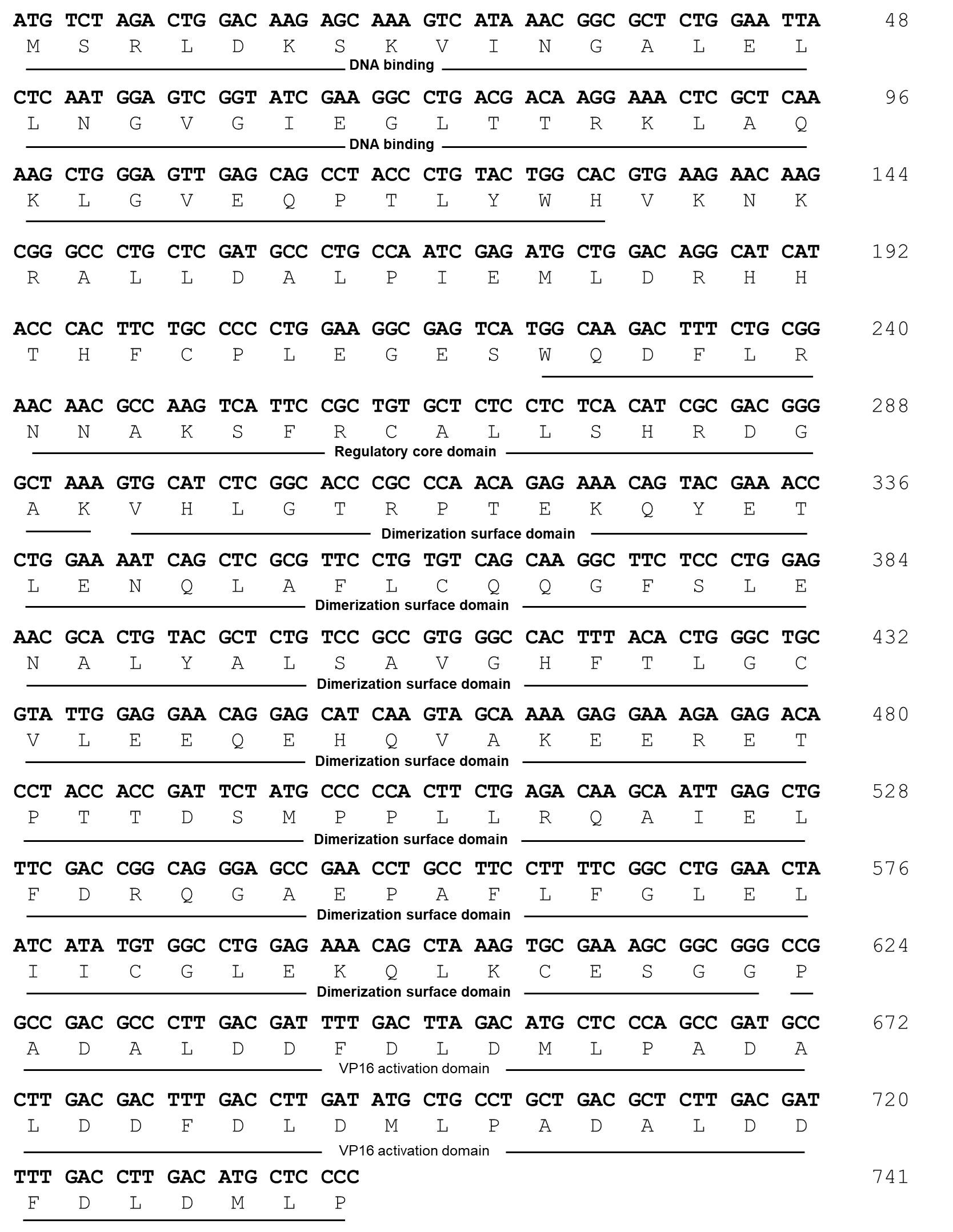


**Figure S1.** The whole rtTA sequence with translation and position of each domain.


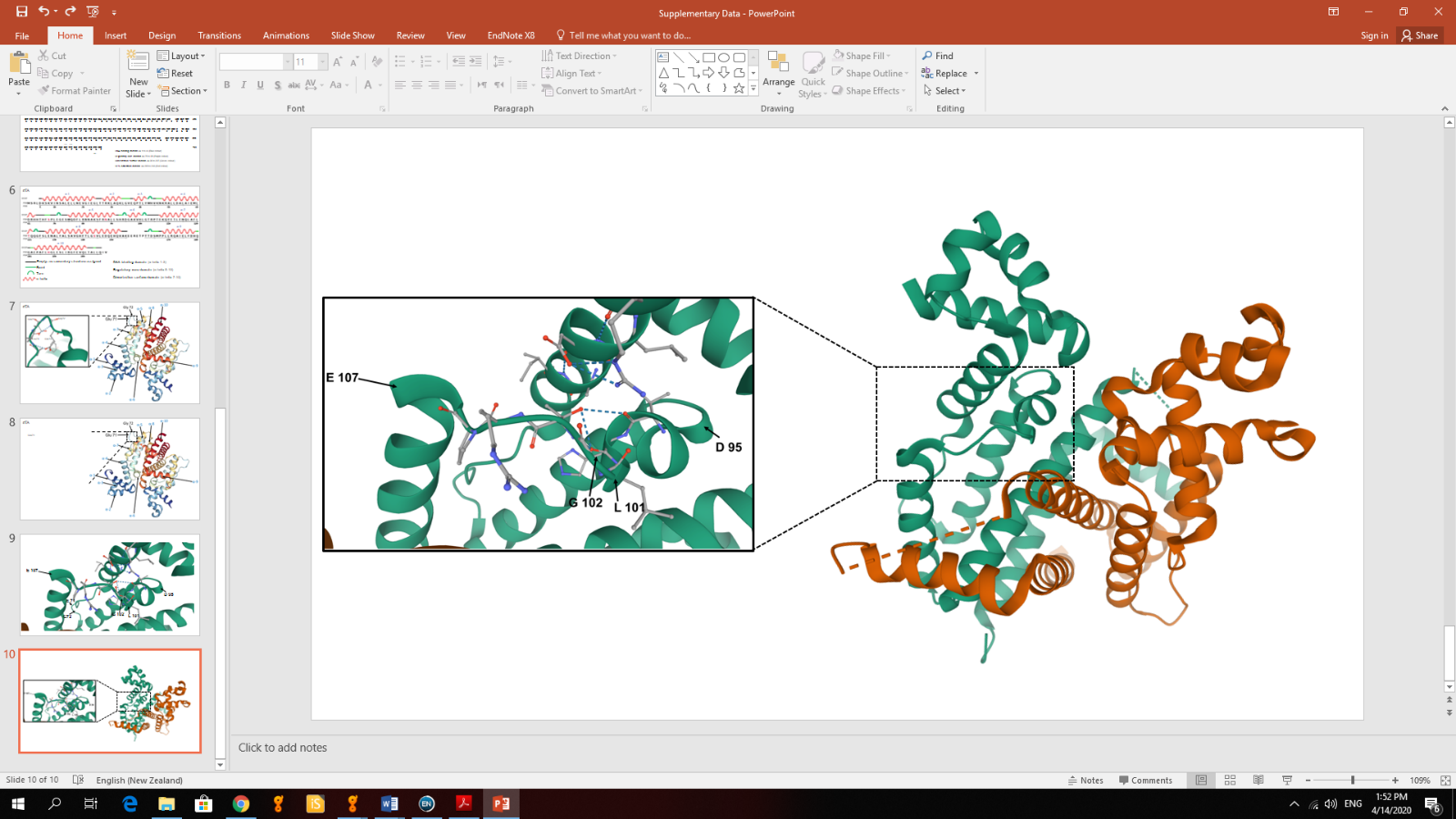


**Figure S2.** Secondary structure of TetR with focus on the high sensitivity region comprising D95, L101 and G102. Modifying the splice site at nucleotide 320 (E107) resulted in a conformational change that decreased TCE-induction upon doxycycline treatment.
